# B Cell Responses in the Development of Mammalian Meat Allergy

**DOI:** 10.3389/fimmu.2020.01532

**Published:** 2020-07-17

**Authors:** Jessica L. Chandrasekhar, Kelly M. Cox, Loren D. Erickson

**Affiliations:** ^1^Beirne B. Carter Center for Immunology Research, University of Virginia School of Medicine, Charlottesville, VA, United States; ^2^Department of Microbiology, Immunology, and Cancer Biology, University of Virginia School of Medicine, Charlottesville, VA, United States

**Keywords:** adaptive immune response, B cells, allergy, meat, IgE, ticks

## Abstract

Studies of meat allergic patients have shown that eating meat poses a serious acute health risk that can induce severe cutaneous, gastrointestinal, and respiratory reactions. Allergic reactions in affected individuals following meat consumption are mediated predominantly by IgE antibodies specific for galactose-α-1,3-galactose (α-gal), a blood group antigen of non-primate mammals and therefore present in dietary meat. α-gal is also found within certain tick species and tick bites are strongly linked to meat allergy. Thus, it is thought that exposure to tick bites promotes cutaneous sensitization to tick antigens such as α-gal, leading to the development of IgE-mediated meat allergy. The underlying immune mechanisms by which skin exposure to ticks leads to the production of α-gal-specific IgE are poorly understood and are key to identifying novel treatments for this disease. In this review, we summarize the evidence of cutaneous exposure to tick bites and the development of mammalian meat allergy. We then provide recent insights into the role of B cells in IgE production in human patients with mammalian meat allergy and in a novel mouse model of meat allergy. Finally, we discuss existing data more generally focused on tick-mediated immunomodulation, and highlight possible mechanisms for how cutaneous exposure to tick bites might affect B cell responses in the skin and gut that contribute to loss of oral tolerance.

## Introduction

Mammalian meat allergy, also known as α-gal syndrome, is among a minority of food allergies that pose a serious acute health risk that induces cutaneous, gastrointestinal, and respiratory reactions in patients after eating non-primate mammalian meat such as beef, pork, or lamb (1, 2). This novel form of food allergy develops worldwide in adults who have tolerated meat consumption for years and is mediated predominantly by IgE antibodies specific for the carbohydrate galactose-α-1,3-galactose (α-gal) that is expressed on cells and tissues of dietary meat (1–11). Allergic reactions in patients range from mild to severe hives, itchiness, nausea, vomiting, and abdominal pain 3–6 h after eating meat (1, 12, 13). Moreover, some patients progress to anaphylaxis after consuming meat, though the reasons for this severe reaction in a subset of patients are unknown. Individuals with α-gal-specific IgE have also reported severe allergic reactions to drugs derived from mammalian cells and tissue that contain α-gal residues (10, 14, 15).

The development of mammalian meat allergy has been linked to tick bites. In the United States, bites from *Amblyomma americanum* (lone star tick) are associated with meat allergy (3). *A. americanum* has been considered a tick species inhabiting the southern and southeastern states. However, the range of *A. americanum* has expanded northward into the northern Mid-western states, north central states, and northeastern Atlantic states as far north as Maine (16–18). One hypothesis for this expansion is that the hosts for lone star ticks, such as white-tailed deer, are increasing in population and migrating northward due to climatic and environmental changes (17, 19). Based on these studies, along with case reports of IgE-mediated anaphylactic reactions to meat cropping up in areas outside the south, it is projected that mammalian meat allergy associated with lone star ticks will increase in future years. Multiple case reports have been further published describing the association between α-gal IgE and meat allergy in Central America (1, 3, 4), Europe (5–7), Australia (2, 20), Asia (8, 9), and South Africa (21). Ticks are endemic in all of these regions yet vary in species. This raises the notion that tick species linked to meat allergy share immune modulating factors that trigger α-gal sensitization.

Unlike other tick-borne diseases caused by viral and bacterial infections that may be prevented by vaccination or antibiotics, there is no treatment to prevent or cure meat allergy. Further efforts are needed to understand the immune mechanisms by which cutaneous exposure to ticks leads to sensitization and the production of pathogenic IgE antibodies. Such efforts would solidify tick bites as the cause of meat allergy and identify new, more specific targets for the treatment and prevention of this food allergy. Here, we review recent progress in studies of the immune reactions in mammalian meat allergy. A particular emphasis is devoted to B cell responses given the important association of α-gal IgE to meat allergy and IgE-mediated drug reactions. We also discuss the features of the α-gal carbohydrate allergen and tick-host interactions that might provide insights into the immune mechanisms that lead to cutaneous sensitization.

## Mammalian Meat Allergy

Allergic reactions against α-gal were first recognized in the United States in 2006 following the FDA approval of cetuximab, a mouse-human chimeric mAb to Epidermal Growth Factor Receptor, for the treatment of advanced bowel and head and neck cancer (22). Clinical trials of cetuximab indicated a low risk of hypersensitivity responses and when reactions in patients did occur they were mild (22, 23). However, as the number of cancer patients being treated with cetuximab increased, a high frequency of hypersensitivity reactions was observed in patients located in the southeastern United States. Studies conducted at the University of North Carolina revealed that severe (grade 3 or 4) reactions occurred in ~22% of cancer patients treated with cetuximab, far higher than the frequency of 3% observed nationally (14). Analysis of pre-treatment serum revealed that the individuals who experienced hypersensitivity reactions had pre-existing IgE that bound to cetuximab. Further work determined that these IgE antibodies were specific to α-gal, a carbohydrate found on the murine portion of cetuximab (10). Numerous case reports of healthy individuals experiencing urticaria, angioedema, or anaphylaxis with no clear cause also came to the attention of physicians during this time (1). These cases occurred in the same southeastern region of the US as the severe hypersensitivity reactions in cancer patients treated with cetuximab. Some individuals indicated that the hypersensitivity responses occurred several hours after consuming red meat, such as beef, pork, lamb, or venison. In many cases, the individuals who experienced these hypersensitivity responses had a history of consuming meat for decades with no adverse reaction (12). Intradermal testing with beef, pork and lamb extracts elicited strong positive results, and oral food challenges ultimately confirmed that eating red meat caused the delayed hypersensitivity responses (1). Further work revealed that α-gal-specific IgE contributed significantly to the allergic response to red meat in these individuals (1). For a more detailed overview of the clinical diagnosis and risk factors for mammalian meat allergy, we refer the readers to two recent reviews (24, 25).

Both cetuximab-induced hypersensitivity and meat allergy cases were restricted to the same geographical area, mainly North Carolina, Virginia, Tennessee, Arkansas, and part of Missouri (14). Three individuals investigating the α-gal syndrome story developed meat allergy and all had been recently bitten by lone star ticks (3). Comparison of serum obtained before and after tick bites in these individuals revealed a significant increase in α-gal-specific IgE titers following tick bites, suggesting that tick bites could drive the production of α-gal-specific IgE antibodies. Further investigation revealed that a large number of patients in the US with meat allergy also had a history of lone star tick bites. Analysis of serum from these meat allergic patients revealed a significant correlation between IgE to α-gal and IgE to *A. americanum* proteins (3, 26), which declines over time in patients who avoid tick bites (27). The total number of annual meat allergy cases in the United States has been estimated to be 5,000 (28). Meat allergy cases associated with α-gal-specific IgE antibodies have also been reported in Australia (2), Brazil (29), France (7, 30, 31), Germany (6), Spain (9), Sweden (5, 32), and Japan (33). Interestingly, subjects with type 1 sensitization to α-gal that clearly tolerate meat consumption have been identified in screening tests (34, 35). Recently, the basophil activation test (BAT) has been shown to distinguish patients with mammalian meat allergy from asymptomatic α-gal-sensitized subjects (36). Why these subjects are asymptomatic after eating meat is unclear but might signify individuals at risk of developing meat allergy.

## Galactose-α-1,3-Galactose

Studies of the biochemical nature and function of α-gal first began more than two decades ago in the xenotransplantation field, as a major target for human anti-pig antibodies is the α-gal epitope expressed on pig organs (37–41). The α-gal epitope is a carbohydrate synthesized on membrane-bound and secreted glycolipids and glycoproteins by the glycosylation enzyme α-1,3-galactosyltransferase (GT) (42). GT catalyzes the last step of α-gal biosynthesis by attaching galactose to the N-Acetyl-D-lactosamine. α-gal is expressed on cells of all non-primate mammals below Old World monkeys, whereas Old World monkeys, apes and humans lack the GT enzyme and thus do not synthesize α-gal epitopes and produce α-gal-specific antibodies (43, 44). All healthy humans naturally produce IgM and IgG antibodies to α-gal beginning in infancy (45), and it is these pre-existing antibodies to α-gal that drive the acute rejection of porcine xenografts (46–49). This production of α-gal-specific antibodies is thought to be driven by the continuous exposure of B cells to α-gal expressed by microorganisms that colonize the gastrointestinal tract (45, 50, 51). α-gal has been found in a variety of bacteria of the human flora, including *Escherichia coli, Klebsiella*, and *Salmonella* strains isolated from human stool and blood, with some strains expressing α-gal on the carbohydrate portion of bacterial lipopolysaccharides (45, 52). Furthermore, several human pathogens express α-gal. These include some that are not tick-borne pathogens, such as *Plasmodium* spp. (53), *Trypanosoma cruzi* (54), *Leishmania* spp. (55, 56), *Mycobacterium marinum* (57, 58), *Aspergillus fumigatus* (59), and *Schistosoma mansoni* (59), as well as others that are tick-borne pathogens, such as *Anaplasma phagocytophilum* and *Borrelia* spp. (60). These findings support the hypothesis that bacteria of normal flora may provide chronic α-gal exposure to B cells for the production of α-gal-specific IgM and IgG antibodies to protect the host against pathogens that express α-gal.

Interestingly, α-gal epitopes have been detected in the gut (32), saliva (29), salivary gland (33), ovaries (61), and cement (62) of several tick species linked to meat allergy. One possibility for the source of this α-gal is residual glycoproteins containing α-gal from a blood meal of non-primate hosts, as α-gal epitopes increase over time in the salivary glands of lone star ticks after blood feeding (63, 64). An alternative explanation is that ticks are able to produce endogenous α-gal. BLAST searches of the putative genes encoding the enzymes for N-glycosylation identified multiple genes in ticks, including *A. americanum*, that were orthologous to those of insects and humans (64). In addition, a number of galactosyltransferase homologs in the *Ixodes scapularis* tick were identified that are involved in the production of α-gal in tick salivary glands and increase after feeding (61). Other possibilities for α-gal sources include bacteria present in ticks. For example, the tick-borne bacteria *Anaplasma phagocytophilum* and *Borrelia burgdorferi* sensu lato express α-gal (60), which may drive α-gal-specific IgE production by increasing α-gal levels in ticks as well as contributing to the immune response. Differences in the microbiome among tick species may determine the presence of α-gal and therefore the tick microbiome is postulated to be a risk factor influencing the development of meat allergy. Further investigating this theory might prove to be challenging, but genomic and transcriptomic sequencing approaches to identify GT genes that are expressed by the microbes living inside ticks may provide supporting evidence for α-gal biosynthesis.

## IgE Immune Responses in Allergy

Allergies involve both innate and adaptive immune responses. The topic of innate immune responses in allergy is not discussed here, but excellent reviews can be found elsewhere (65–67). The initial encounter with allergen occurs at the epithelial barrier such as the lung epithelia for air allergens, the gut epithelia for food allergens, and the skin epithelia for insect venom allergens. Epithelial cells and tissue resident innate immune cells are known to play an important role in inducing cytokines that favor host T helper 2 cell (Th2) responses. In addition, dendritic cells (DC) play an important role in initiating the adaptive immune response to allergen. DCs present antigen to T cells and provide costimulatory signals that are vital to activating these T cells. Upon antigen binding, tissue resident DCs traffic to the draining lymph nodes, where they present antigen to T cells and drive Th2 cell differentiation (68–71). Early work on IgE regulation demonstrated an important role for Th2 cells, as deletion of Th2 lineage-defining transcription factors such as STAT6 or GATA3 reduced IgE production (72–74). In addition, deletion of the canonical Th2 cytokine IL-4 reduces IgE. Recent studies have demonstrated that IL-4^+^ T follicular helper (Tfh) cells, essential for germinal center (GC) formation in secondary lymphoid organs, are required for IgE production (75–80). Moreover, a rare subset of IL-4^+^ Tfh cells that produces IL-13 has been identified and induces high-affinity IgE to allergens (81).

Although little is known about how tick bites drive the IgE response of allergen-specific B cells, multiple studies have shown that Th2 responses are induced by tick saliva—considered by some in the field to be a special kind of venom (82). Tick-derived factors that drive Th2 responses are thought to facilitate transmission of tick-borne pathogens that would otherwise be neutralized by Th1 cell-mediated responses. Human and mouse CD4^+^ T cells produce Th2 cytokines in response to tick saliva, including IL-4 and IL-13, with reduced production of the Th1 cytokine IFNγ (83–89). Many of the factors in tick saliva thought to play a key role in shaping the innate immune response for inducing Th2 responses include prostaglandins, sphingomyelinase, and cysteine protease inhibitors (90–93). These factors could influence the pattern of CD4^+^ T cell responses by acting on antigen presenting cells to create a cytokine milieu that favors the development of Th2 cells while suppressing Th1 differentiation (94). In addition, these factors could directly act on B cells to enhance class switch recombination to IgE. This notion is supported by increased production of IgE in mice treated with the prostaglandin, PGE2 (95), and from murine B cells stimulated with LPS plus IL-4 in the presence of PGE2 (96–99). However, non-polarized or mixed Th1 and Th2 responses are also observed following tick bites (100, 101). These differences could reflect the tick species, the community of microbiota living inside the ticks, or temporal changes in the tick saliva-derived factors throughout the duration of feeding, including protein changes in the saliva of lone star ticks as previously reported (102–105). Meat allergic patients show a Th2-skewed profile, as measured by higher α-gal IgE, IgG1 and IgG4 levels (106). This suggests that tick bites of affected individuals drive sensitization to α-gal by promoting Th2-mediated immune responses, possibly through induced antibody class switching by tick salivary PGE2, leading to an increase in the frequency of B cells producing α-gal-specific IgE (107).

### B Cell Differentiation Pathways to IgE

Recent studies using IgE-reporter mice support two differentiation pathways for the generation of IgE-secreting plasma cells (PC). The first is that IgE^+^ PCs develop from the responding progeny of GC-derived IgE^+^ B cells and IgE^+^ memory B cells that are directly derived from IgM^+^ naïve B cells (108). The second is that IgE^+^ PCs develop through indirect isotype switching from the responding progeny of GC-derived IgG1^+^ B cell intermediates and from IgG1^+^ memory B cells following antigen exposure (109, 110). The indirect isotype switching pathway results in IgE that has a higher affinity for antigen due to greater levels of somatic hypermutation in positively selected memory B cells and PCs. However, it should be noted that GC-independent IgE^+^ B cell responses have also been observed through mechanisms that remain unknown (111). Further work is needed to elucidate the processes of GC-independent differentiation of IgE-producing PCs and to determine its contribution to the development of pathogenic IgE in allergy.

In humans, the biology of IgE-expressing B cells remains enigmatic, mainly because of their scarcity and the lack of robust assays that allow for comprehensive immunophenotyping of rare IgE^+^ B cells. The half-life of IgE in serum is short (i.e., 2–3 days), but the capacity to regenerate allergen-specific IgE and develop food allergies are lifelong in the majority of patients. This suggests the existence of long-lived, allergen-specific memory B cells (MBC) established at the time of sensitization, which replenish the short-lived IgE-secreting cells on allergen re-exposure (112). IgE^+^ MBCs are found at extremely rare frequencies in the blood of healthy donors, atopic donors, and donors with food allergy (111, 113, 114). Some studies suggest that the reservoir of IgE-producing cells instead resides in MBCs of a non-IgE isotype (IgM^+^ or IgG^+^) (112). Class-switching to IgE in these memory B cells is thought to take place in germinal centers (115), with indirect isotype switching of IgG-expressing B cells to IgE found more prevalent both in healthy and allergic subjects compared to direct IgE switching (116, 117).

A number of studies have examined the sequences of Sμ-Sε switch regions in allergic individuals and found that these regions contained Sγ switch region remnants, suggesting that IgE was generated through indirect isotype switching (111, 117–120). Moreover, common lineages in IgG1^+^ and IgE^+^ B cells have been found in human subjects when analyzing the repertoire of the rearranged immunoglobulin genes (117), supporting the view that IgE was generated through an indirect switching pathway. In contrast, studies analyzing somatic hypermutation and the correlation between different antibody isotypes in patients with allergies to house dust mite and the fungus *Alternaria alternata* suggest that IgE is derived through direct switching from IgM to IgE (121). Moreover, a population of unswitched, IgM^+^ MBCs that have undergone somatic hypermutation have been described in humans (122–124). These IgM^+^CD27^+^ cells are long-lived (125), and rapidly differentiate into antibody-secreting cells following stimulation (126, 127). Limitations in human studies, including low frequency of IgE^+^ cells in the circulation and inability to assess class switch recombination *in vivo* following allergen challenge, have so far prevented the resolution of these conflicting data and the contribution of direct and indirect switching in the production of pathogenic IgE.

Given these limitations in human studies, many groups have turned to mouse models to study the contribution of direct vs. indirect switching to IgE production. Similar to the methods used in the human studies noted above, Sγ1 fragments within Sμ-Sε switch regions have been detected in mice (109). This study found that the frequency of Sμ-Sε switch regions containing Sγ1 fragments increased with repeated immunization, as did the affinity of IgE antibodies for antigen, suggesting that indirect switching was necessary for the production of IgE^+^ B cells with high-affinity for antigen. This conclusion was further supported by reduced levels of somatic hypermutation and affinity maturation of IgE antibodies produced by mice that were unable to produce IgG1. These data conflict with an earlier study (128), in which mice with impaired switching to IgG1 exhibited no defect in the primary or secondary IgE response to infection with the helminth, *Nippostrongylus brasiliensis*, or immunization with the protein antigen, 4-hydroxy-3-nitrophenylacetyl-ovalbumin (NP-OVA). However, the affinity of the IgE antibodies generated in this study was not assessed, and further characterization of IgE produced in these mouse models could resolve the discrepancy between these two studies.

### Longevity of IgE^+^ Plasma Cells

Another key aspect of allergic memory that remains unclear is the development of long-lived IgE-secreting PCs. Long-lived IgG-secreting PCs have been detected in both humans and mice, and play a vital role in maintaining humoral immunity (129). These cells are maintained in survival niches, often in the bone marrow, continuously secreting protective IgG antibodies for years (130). Murine studies have been employed to investigate the development of long-lived IgE-secreting PCs, as the bone marrow and other lymphoid organs can be easily probed for these cells. It has been shown that long-lived IgE^+^ PCs are generated during the allergic response to OVA (131). IgE-secreting PCs were detected in the bone marrow of immunized mice a month after challenge, far beyond when short-lived PCs in the draining lymph node have declined. These bone marrow IgE^+^ PCs were resistant to treatment with cyclophosphamide, indicating that these cells had exited the cell cycle which is a hallmark of long-lived PCs (132). Experiments in IgE-reporter mice also support the notion that long-lived IgE^+^ bone marrow plasma cells are generated during the allergic response (108, 133). Allergen-specific IgE produced from bone marrow PCs was capable of inducing anaphylaxis in mice with chronic house dust mite exposure (133). In contrast, only short-lived IgE^+^ PCs, found in the draining lymph node, were detected in Verigem mice following challenge with allergen (134). The use of different genetic modifications of the IgE locus, adjuvants, and routes of sensitization in the murine studies detailed above may account for the conflicting conclusions about the development of long-lived IgE^+^ PCs.

Several clinical observations suggest that long-lived IgE^+^ PCs develop in patients with allergy. Low levels of allergen-specific IgE are chronically found in the serum of atopic individuals, even in the absence of exposure to allergen (131). Additionally, transfer of peanut allergy from donor to recipient following bone marrow transplant has been reported multiple times (135–137), suggesting that the bone marrow of donor allergic patients contains long-lived, allergen-specific IgE^+^ PCs. Monoclonal antibody-mediated depletion of IgE^+^ B cells in atopic patients reduces serum IgE but not to baseline levels, thereby indicating enduring cellular sources of IgE production (138, 139). Finally, IgE^+^ PCs were recently found in bone marrow of three cat-allergic patients that secreted detectable levels of total and cat dander-specific IgE and caused mast cell degranulation (133). In summary, these findings provide evidence that human IgE^+^ PCs reside in the bone marrow and secrete IgE capable of triggering an allergic response in at least some atopic patients. Efforts to further characterize these cells and resolve whether IgE^+^ PCs in the bone marrow is broadly shared in allergy will be needed to devise new treatment strategies to target the cellular sources of allergen-specific IgE.

## B Cell Responses in Meat Allergy

In meat allergic patients, the mechanisms by which tick bites sensitize individuals to tick antigens such as α-gal are unknown. Given that α-gal exposure alone does not induce an IgE response (140, 141), two hypotheses have been proposed for the production of α-gal-specific IgE. One is that the presence of α-gal in the context of tick-derived immunomodulatory factors cause antibody class switching to IgE in pre-existing α-gal-specific IgM^+^ or IgG^+^ B cells. The other is the presentation of α-gal to the immune system following a tick bite activates mature α-gal-specific B cells to differentiate into memory cells which later undergo class switch recombination to IgE. Given the ethical aspects of exposing humans to ticks, many groups have turned to animal models to study the underlying immune mechanisms of tick-borne diseases.

### IgE Production in Mice That Develop Meat Allergy-Like Disease

**Figure 1:**
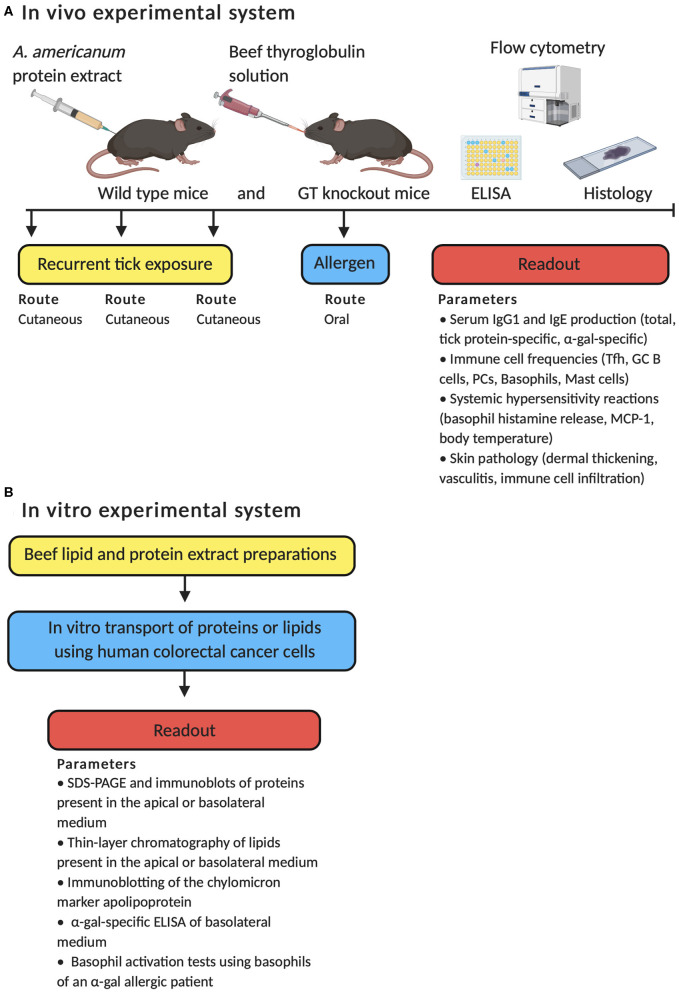
Schematic overview of the *in vivo* and *in vitro* experimental approaches employed to test the immune mechanisms by which α-gal induces an IgE-mediated immune response. **(A)** Development of meat allergy to *A. americanum* in wild type mice and mice deficient in the α-1,3-galactosyltransferase (GT) gene. **(B)** α-gal from meat is transported across enterocytes and activates basophils through α-gal-specific IgE.

The host immune response to *A. americanum* was first tested in guinea pigs (142). This study found that guinea pigs acquired resistance to the recurrent feeding of lone star ticks, which resulted in dermal cellular infiltration in lesions around the tick bites with the accumulation of granulocytes, including basophils. However, *A. americanum* has not yet been used to test IgE-mediated hypersensitivity responses and the underlying immune mechanisms of disease development. Recent work from our group shows that a mouse model can recapitulate features of meat allergy in humans (143). In this study, repeated cutaneous exposure to lone star tick protein antigens, to mimic the natural route of tick bites, leads to the development of red meat allergy-like disease in mice (Figure 1A). The disease is characterized by high titers of serum IgE antibodies, local inflammation at the site of antigen exposure (e.g., dermal thickening; vasculitis and muscle fiber atrophy; infiltration of granulocytes, lymphocytes, and plasma cells within the dermis and hypodermis), and a systemic hypersensitivity response in tick-immunized mice after eating meat (e.g., increased frequencies, activation, and histamine release of basophils in blood; greater serum mast cell protease-1 levels). Although our report used beef thyroglobulin to induce *in vivo* allergic hypersensitivity reactions, other *in vitro* systems have used α-gal bound to beef proteins or lipids to test allergic reactions. Román-Carrasco et al. reported that only α-gal bound to lipids, but not to proteins, crossed a monolayer of intestinal cells and activated basophils from a patient with meat allergy (Figure 1B) (144). Why α-gal-containing beef protein enhanced basophil activity in our study but had no effect in the referenced report is unclear; however, different experimental systems were used with distinct assays to measure mouse and human basophils. As such, a direct comparison cannot be made that controls for other confounders.

Our model is unique in that wild type mice and mice deficient in the α-1,3-galactosyltransferase gene (GT KO) are used. Wild type mice synthesize α-gal epitopes and therefore naturally do not produce α-gal-specific antibodies because of immune tolerance, whereas GT KO mice are not immune tolerant to α-gal. After tick antigen exposure, both wild type and GT KO mice produce tick protein-specific IgE antibodies but only GT KO mice produce α-gal-specific IgE. Interestingly, dogs, which also synthesize α-gal, have detectable levels of α-gal-specific IgM and IgG that increase after tick infestation without affecting IgE (60). These findings are consistent with our results in wild type mice that do not produce α-gal-specific IgE after cutaneous exposure to tick antigens, suggesting that immune tolerance may prevent α-gal sensitization. Both local inflammation in the skin around antigen exposure and hypersensitivity responses are greater in GT KO mice compared with wild type mice, which are phenotypes analogous to human meat allergy (143). Importantly, the development of an IgE response in mice is dependent on cutaneous tick exposure as other routes of tick antigen exposure fail to induce IgE, which is in keeping with evidence in humans that sensitization to α-gal requires skin exposure. Consistent with the above findings, it was also demonstrated that subcutaneous injection of saliva obtained from the Brazilian *Amblyomma sculptum* tick induced α-gal-specific IgE production in GT KO mice (29). This study provides additional evidence for a link between mammalian meat allergy and bites from other tick species.

Our work further demonstrated that cutaneous exposure to tick antigens induced T follicular helper cells and GC B cells in the skin draining lymph nodes. CD4^+^ T cell help was required for sensitization to produce IgE antibody responses, as total and tick-specific IgE levels were reduced in mice depleted of CD4^+^ T cells or when treated with a CD154 mAb to block GC B cell formation (143). Interestingly, the development of host resistance to *A. americanum* first reported in guinea pigs was associated with germinal center formation in the lymph nodes draining tick feeding sites (142).

**Figure 2:**
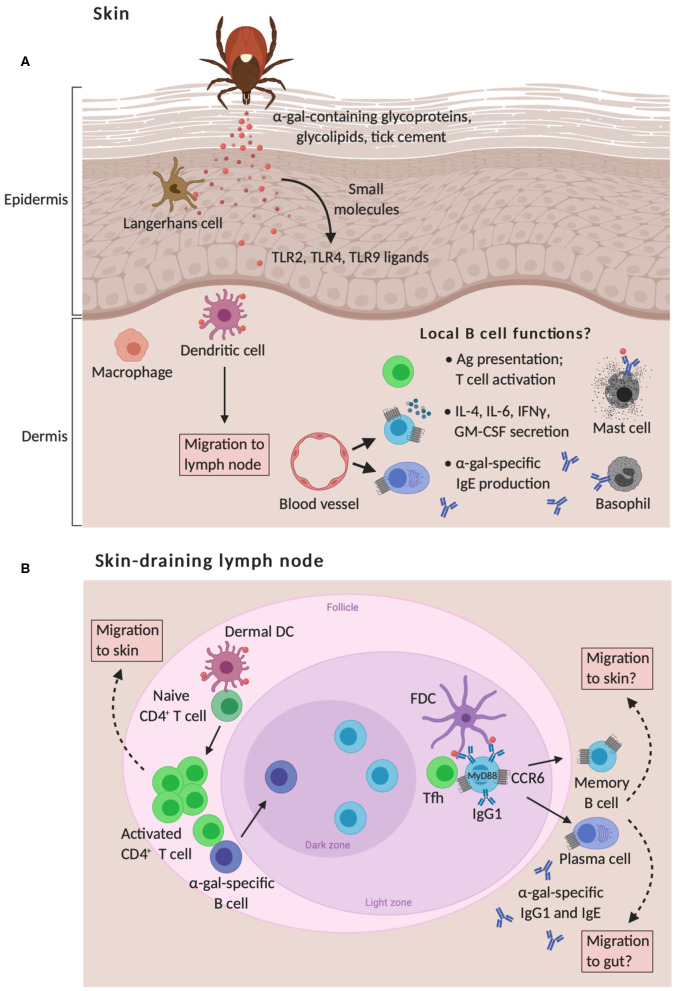
Proposed model of skin-associated B cell functions in α-gal sensitization from tick bites. **(A)** The skin is comprised of the epidermis and the dermis that are separated by a basement membrane. Langerhans cells (LC) and dermal dendritic cells (DC) are capable of responding to cutaneous exposure of tick antigens, such as glycoproteins, glycolipids, and tick cement that contain α-gal moieties, which leads to the migration of these cells to the skin-draining lymph nodes and allergen sensitization. This response is also shaped by the migration of B cells to inflamed skin around the tick bite, which may contribute to allergic responses by presenting antigen to T cells, secreting pro-inflammatory cytokines, and secreting IgE antibodies that trigger mast cell and basophil activation. To date, the role of skin-associated B cells in meat allergy is unknown. **(B)** Naïve T cells are primed via presentation of tick antigens by LCs and dermal DCs within skin-draining lymph nodes. Activated CD4^+^ T cells subsequently traffic to the skin through blood and lymphatic vessels. Cognate T cell help provided by T follicular helper (Tfh) cells to α-gal-specific B cells leads to germinal center responses, positive clonal selection of B cells via recognition of native antigens retained by follicular dendritic cells (FDC), and the development of memory B cells and plasma cells that express skin- and gut-homing CCR6 receptors. IgE production to cutaneous tick antigens functions through a B cell-intrinsic myeloid differentiation primary response protein 88 (MyD88) pathway and indirect class switching of IgG1^+^ B cells to IgE.

Tick protein antigens had an adjuvant effect in our mouse model for IgE production, suggesting that, in addition to allergen exposure, the presence of other immunomodulatory factors is required for sensitization. Analysis of lone star tick protein antigens using a commercial toll-like receptor (TLR) screen indicated that ligands for TLR2, TLR4, TLR5, and to a lesser degree, TLR9, were present. Notably, all of these TLRs signal through the protein encoded by the myeloid differentiation primary response gene 88 (MyD88) (145). We found that MyD88 expression was required for the IgE response to cutaneous tick antigen exposure, as total and tick-specific IgE production was reduced in the absence of MyD88. Moreover, reduced levels of IgE antibodies were observed in mice that conditionally delete MyD88 in B cells that have class switched to IgG1. Together, the above findings support a model where allergen-specific B cells are stimulated through the MyD88 signaling pathway in germinal centers to produce IgE, with indirect class switching of IgG1-expressing B cells to IgE (Figure 2). MyD88 has been shown to be involved in Th1 cell responses (145); however, more recent work has demonstrated that MyD88 signaling also plays a role in Th2 cell-mediated responses (146–148). Because signaling through TLR2, TLR4, and TLR9 has modulatory effects on the mucosal immune system and has been shown to induce IgE production (149–151), it is possible that *A. americanum*-derived TLR2-, TLR4-, and TLR9-activating small molecules exert host adverse effects by promoting skin inflammation at the tick bite site. This might aid in the activation of pre-existing α-gal-specific B cells and sensitization to tick antigens, although this has yet to be formally tested. This notion is supported by recent observations that several allergens contain TLR ligands and activate MyD88 signaling in innate and adaptive immune cells, suggesting an important role of distinct pathogen-associated molecular patterns in the development of allergy (148, 152–155). However, TLRs are not the only immune receptors to signal through MyD88. Members of the IL-1 receptor family, including IL-1R, IL-18R, and IL-33R, also signal through MyD88 (156). These observations raise the possibility that signals through these receptors on innate and adaptive immune cells could be used to drive allergic sensitization to tick antigens and require further investigation. A new zebrafish animal model for the study of allergic reactions in response to tick saliva and red meat consumption has recently been developed that might be useful in this pursuit (157). Zebrafish do not express α-gal in their tissues and produce α-gal-specific IgM, similar to humans. Interestingly, allergic reactions in zebrafish manifest only when previously exposed to tick saliva and are associated with tissue-specific TLR-mediated responses in Th1 and Th2 cells.

Small molecules within tick saliva are known to exhibit immunomodulatory effects on a wide variety of both skin resident and infiltrating cell types, facilitating local and systemic responses (158). For example, salivary molecules from multiple tick species have been identified that impede natural host hemostatic responses to tick bite-induced tissue damage, promoting vasodilation and countering platelet aggregation, angiogenesis, and the coagulation cascade (159–165). The saliva from ticks also has anti-inflammatory effects that might favor a Th2 immune response. For example, saliva from *Rhipicephalus appendiculatis* ticks prevents inflammation by impairing the secretion and function of certain soluble mediators, such as histamine (166). Saliva from *Rhipicephalus sanguineus* ticks inhibits the migration of dendritic cells into and out of the skin, reducing antigen presentation to T cells in draining lymph nodes (101). Saliva from the tick *Ixodes ricinus* contains proteins of the lipocalin family that inhibit the host inflammatory response *in vivo* by decreasing the number and activation of neutrophils in the skin around the tick bite site (167). Finally, gene expression profiling of cutaneous bite-site lesions in mice from the tick *Ixodes scapularis* shows increased expression of anti-inflammatory molecules such as IL-10, SOCS1, SOCS3, and the regulatory T cell-associated Forkhead box P3 (FOXP3) transcription factor early (<3 h) after tick attachment (100). However, by 12 h after tick attachment, an inflammatory response develops as measured by the upregulation of transcripts for IL1β, IL-6, CCL2, and CCL7, that facilitate local recruitment of neutrophils, degranulation of mast cells, and muscle necrosis at the tick bite site (168). These studies suggest that there is at least some degree of pro- and anti-inflammatory response mounted following tick bites. Interestingly, with the exception of *I. ricinus*, none of the above-mentioned tick species are linked to mammalian meat allergy. It is therefore possible that microbial ligands within tick saliva might be differentially expressed and recognized by host immune cells during tick bites.

How the skin route of tick exposure initiates allergic sensitization to food allergens such as α-gal and evades oral tolerance is poorly understood. The skin is an important barrier organ and integral component of the immune system that protects the body from external insults, but it is also a target for allergic disease. Skin barrier disruption and cutaneous exposure to food allergens in particular are strongly associated with food allergy (169–171). Thus, it has been hypothesized that sensitization to food allergens can occur through damaged skin, leading to Th2 immune responses in the draining lymph node and the production of systemic allergen-specific IgE antibodies that characterize allergic disease. There is limited knowledge of the role for skin-associated B cells in this process, where they may perpetuate local inflammation by enhancing cutaneous allergen-specific IgE titers. In many chronic inflammatory human diseases that have been presumed to be driven by T cells, such as atopic dermatitis (172), psoriasis (173), pemphigus (174), cutaneous lupus (175–177), allergic contact dermatitis (178), and scleroderma (179), B cells accumulate in inflamed skin. Many roles for B cells have been postulated in these diseases beyond their contributions to pathogenic antibody production, including their ability to present antigen and secrete pro-inflammatory cytokines, like IL-6, GM-CSF, IFN-γ, all of which promote local inflammation (180). In addition, cellular compartments called tertiary lymphoid structures (TLS) that support interactions between skin B and T cells are found in sites of chronic inflammation (174, 181). These structures are variable and can be mature, forming germinal centers that might facilitate B cell function, or immature, that do not form GCs and therefore may not support B cell function. Mature TLS in the skin might provide a local microenvironment for skin-associated B cells to promote inflammation by enhancing pathogenic antibody production and T cell activation. In contrast, B cells in skin can produce inhibitory factors that suppress activation of other leukocytes. B regulatory cells that produce IL-10 have been found in skin and limit cutaneous inflammation in mouse models of psoriasis and contact hypersensitivity (182–184). Thus, skin-associated B cells are a heterogenous population of discrete B cell subsets that can drive or suppress inflammatory responses. Interrogating B cells quantitatively in disease tissue, what they secrete, and what cells these B cells physically interact with are key approaches that could help predict a patient's response to tick bites and establish new immune-based therapies to block allergic sensitization.

Our own observations of inflamed skin at the site of tick antigen exposure demonstrate the accumulation of leukocytes, with greater numbers of CD4^+^ T cells, B cells, and plasma cells found in immune GT KO mice compared to naïve controls (143) and unpublished observations. Strikingly, these B cells express high levels of the chemokine receptor CCR6 and have an activated phenotype as measured by increased expression of MHCII and CD86 levels. Increased frequencies of CCR6^high^ B cells are also found in skin draining lymph nodes and Peyer's patches in the gut of the same mice. Expression of CCR6 on B cells is associated with migration both to Peyer's patches, major sites of circulating B cell homing and activation by intestinal antigens (185, 186), and from skin draining lymph nodes to the skin (187). CCR6 and its ligand, CCL20, also can contribute to skin and gut homing of T cells (188–190). These data support the notion that tick antigen-specific CCR6^high^ B cells are generated in the skin draining lymph node before trafficking to inflamed skin and to the gut where they may encounter α-gal and further differentiate into IgE-secreting cells (Figure 2). Ongoing studies are aimed to formally test this hypothesis. An implication of this hypothesis is that if the skin inflammation mediated by tick bites can be prevented, α-gal-specific IgE production could be impaired and the incidence of meat allergy might be decreased. This represents a substantial shift in thinking concerning the target organ for food allergy prevention.

### B Cell Responses in Human Meat Allergic Patients

Our findings linking cutaneous lone star tick exposure and α-gal-specific IgE production with meat allergy development in mice prompted us to ask whether distinct IgE^+^ B cell phenotypes are enriched in individuals with meat allergy following tick bites. Our recent work using high-dimensional mass cytometry indicates that four main B cell subsets are significantly enriched in the blood of meat allergic patients compared with non-meat allergic controls (191). These B cell subsets share high expression levels of CCR6 in keeping with findings from our mouse studies. They do not share typical characteristics of classical isotype-switched memory B cells that express IgG and high levels of CD27 (123, 126), with B cells expressing IgM or IgD and low or high levels of CD27. Testing the capacity of these B cell subsets to secrete antibodies following *in vitro* stimulation demonstrated that such cells undergo isotype-switching and produce α-gal-specific IgE in patients with meat allergy but not in healthy control subjects (191). The specific role of direct vs. indirect class switch recombination to IgE was not tested in this study, and further work is necessary to determine the mechanisms of clonal selection and the ontogeny of α-gal-specific B cells. These B cells may include a novel population of memory cells lacking CD27 expression found in the blood that are prone to differentiate into IgE-secreting cells (192, 193). Memory B cells displaying low to negative CD27 expression are also found in human tissues near epithelial surfaces (194, 195). Early work in human adults identified peripheral blood memory B cells that express IgM with or without IgD, or express IgD only (122, 123, 196–198); these cells show somatically mutated IgV genes suggesting the involvement of T cell help for their development. Together, the above findings support a novel B cell signature in meat allergic subjects that associates with α-gal-specific IgE production, which may play a role in the pathogenesis of this food allergy.

This B cell signature might help explain the positive association of subjects who do not express the blood group B antigen among those with mammalian meat allergy (5, 199). Because the molecular structure of α-gal is closely related to the B antigen (45), it has been hypothesized that patients expressing the B antigen are tolerant to α-gal and therefore protected from allergic sensitization to α-gal. Patients with the B antigen produce less α-gal-specific IgE compared with those without the B antigen (199, 200), and is associated with reduced Th2-like IgG1 and IgG4 antibody responses to α-gal (106, 200). We theorize that tick bites present α-gal to the immune system in a context that elicits the activation and persistence of memory B cells to α-gal from which α-gal-specific IgE is derived under control of T cell help. Although subjects who express the B antigen can be sensitized to α-gal and develop meat allergy, those who do not express the B antigen may have stronger Th2 responses that cause recall of distinct B cell memory subsets to α-gal. This possibility may also affect the increased susceptibility to infectious diseases caused by pathogens carrying α-gal on their surface, such as malaria and tuberculosis, in individuals with the B antigen (58). Identifying the immune profile of CD4^+^ T cell subsets from meat allergic patients with and without B antigen and the functional capacity of each subset to induce activation of B cell subsets could help instruct the relationship between memory B cells, blood group B antigen, and α-gal sensitization. This knowledge could help to shape strategies for improved vaccines for human pathogens that display α-gal. Several α-gal-based vaccines have enhanced humoral and/or cell-mediated responses to *Leishmania* and *Trypanosoma cruzi* that have membrane-expressed α-gal epitopes, using the GT KO mouse model (54–56). Additionally, this information might provide clues how α-gal can increase the immunogenicity of viral vaccines designed to express α-gal epitopes, including flu vaccine, for effective targeting of the vaccine to antigen presenting cells, thereby increasing its efficacy (201).

Whether the circulating B cell subsets found in meat allergic patients migrate to inflamed skin during tick bites is unknown. In studies examining immune cells locally in skin at the lone star tick bite site, migratory B cells and T cells were implicated in meat allergic patients within 2 days after tick bites (202). Comparison of serum IgE titers from a meat allergic subject obtained 5, 14, and 35 days after tick bites showed increased total and α-gal-specific IgE (3). These findings indicate that α-gal IgE is rapidly induced following tick bites, suggesting a link between cutaneous exposure to α-gal coupled with inflammatory signals and the activation of pre-existing α-gal-specific B cells in meat allergic subjects. Based on these findings, we hypothesize that skin-associated B cells at the tick bite site contain a highly expanded, α-gal-specific B cell population that contributes to the initiation and maintenance of meat allergy via production of IgE. Ongoing human and mouse studies by us and other groups should inform whether B cells locally produce IgE to promote inflammation, their developmental pathways, and how these cells can be targeted therapeutically.

## Concluding Remarks and Open Questions

Within the past few years, we have started to gain new information about how specific host- and tick-derived molecules shape the adaptive immune response to ticks associated with mammalian meat allergy. Continued research into the specific mechanisms by which tick bites trigger α-gal sensitization is important for devising new therapeutic strategies to treat and prevent this allergy. The skin contains many innate and adaptive immune cell types including DCs, macrophages, mast cells, innate lymphoid cells, T cells, and B cells. B cells have a major role in protection against pathogens through antibody production but also through antigen presentation and cytokine production. B cell function also influences the activity of other immune cell types. In this aspect, knowing how B cells respond to tick-derived antigens and immunomodulatory factors and migrate to the skin and gut will be important for controlling tick-borne allergies. Several major questions still remain that need to be addressed to generate a complete picture:

1. The contribution of skin-associated B cells to the generation of α-gal-specific IgE. Since B cells are expanded in inflamed skin in several other allergy and autoimmune diseases, this knowledge might be broadly applicable to fundamental B cell biology in several disease contexts beyond meat allergy.
2. Determining the relationships between α-gal-specific IgM and IgG antibodies, and the B cell populations that produce them, to α-gal-specific IgE. Evaluating the antibody repertoire in patients with meat allergy by analyzing B cell receptor clonality, use of Ig heavy chain variable region genes, affinity maturation to α-gal, and isotype use would provide a more detailed understanding of how α-gal IgE antibodies are generated.
3. Understanding how cutaneous exposure to α-gal and other tick-derived molecules leads to the loss of oral tolerance. This will require development of models that allow testing of specific molecules identified in tick saliva and the capacity to probe innate and adaptive immune cell dynamics in a temporal manner in the skin and GALT.
4. Deciphering why certain tick species associate with mammalian meat allergy while others do not. Ideally this should integrate microbiome analyses with proteomics of tick saliva and gastrointestinal tract to identify bacterial compositions and the expression of α-gal.

## Author Contributions

JC, KC, and LE wrote and edited the manuscript. All authors contributed to the article and approved the submitted version.

## Conflict of Interest

The authors declare that the research was conducted in the absence of any commercial or financial relationships that could be construed as a potential conflict of interest.
